# *Lactobacillus acidophilus*-Induced Endocarditis and Associated Splenic Abscess

**DOI:** 10.1155/2020/1382709

**Published:** 2020-04-04

**Authors:** Muhammet Ozer, Suleyman Yasin Goksu, Ali Shahverdiani, Muhammad Mustafa

**Affiliations:** ^1^Department of Internal Medicine, Capital Health Regional Medical Center, Trenton, NJ, USA; ^2^Department of Internal Medicine, University of Texas Southwestern Medical School, Dallas, TX, USA; ^3^Division of Interventional Cardiology, Capital Health Regional Medical Center, Trenton, NJ, USA

## Abstract

*Lactobacillus acidophilus* is a rod-shaped, Gram-positive bacterium generally found in the normal flora of the oropharynx, gastrointestinal, and genitourinary tracts. It is commonly known as nonpathogen in the human body. Endocarditis due to *Lactobacillus* is rarely encountered and associated with impaired immunity. Splenic abscess is also an uncommon infection that classically results from endocarditis or another source of hematogenous seeding. Here, we present the case of bioprosthetic aortic valve endocarditis and associated splenic abscess caused by *Lactobacillus acidophilus*. The source of the *Lactobacillus bacteremia* should be investigated because of the risk of life-threatening conditions. Most of the time, identifying *Lactobacillus* species is challenging and can cause a delay in diagnosis and timely treatment. Especially in patients who have significant underlying clinical conditions, physicians should consider *Lactobacillus* species as a causative microorganism.

## 1. Introduction

*Lactobacillus acidophilus* is a facultatively anaerobic, rod-shaped, Gram-positive bacterium generally found in the normal flora of the oropharynx, gastrointestinal, and genitourinary tracts and rarely causes infection in humans. *Lactobacillus acidophilus* is also used as a probiotic for specific conditions due to its effects on the stimulation of the immune system and the ability to prevent the colonization of the pathogenic microorganisms in colonic and genitourinary mucosa [1]. Infective endocarditis is a relatively rare disorder with an estimated incidence of 3–10 cases per 100 000 population per year [2]. Endocarditis due to *Lactobacillus* species are rarely encountered and is associated with impaired immunity and severe comorbidities [3]. In the literature, the mortality rate for anaerobic organisms related infective endocarditis ranges from 21 to 43% [4]. In a prospective study with 2491 patients, Kestler et al. found 18% mortality rate during admission [5]. Cannon et al. reported that *Lactobacillus* species induced infective endocarditis associated with a 30% mortality rate [6]. *Lactobacillus casei* and *Lactobacillus rhamonsus* are the most commonly reported species, whereas *Lactobacillus acidophilus* was reported only in a few case reports [7–9]. It is crucial to determine the infecting species and its sensitivity profile so as to provide the most effective therapeutic regimen and obtain a favorable treatment outcome [10].

Splenic abscess is also an uncommon infection that classically results from endocarditis or another source of hematogenous seeding. We reported a rare case of infective endocarditis and splenic abscess caused by *Lactobacillus acidophilus* in a patient who has poorly controlled diabetes mellitus, bioprosthetic aortic valve, and history of IV drug use. In this report, we give attention to a rare presentation of this rare organism. To the best of our knowledge, this is the first *Lactobacillus acidophilus*-induced concurrent endocarditis and splenic abscess case in the current literature.

## 2. Case Description

**Figure 1:**
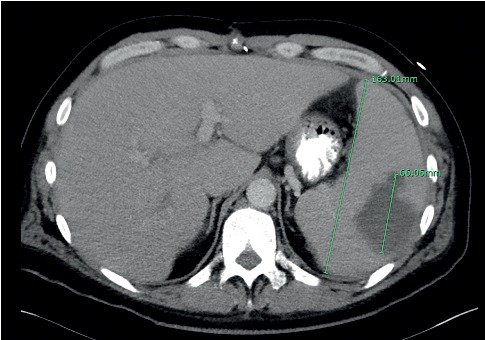
CT scan of the abdomen and pelvis with contrast showing splenic infarct with large splenic abscess collection in size of 66.05 mm and the enlarged spleen in size of 163.01 mm.

The patient is a 42-year-old Caucasian male with a past medical history of uncontrolled diabetes mellitus, IV drug use, and bioprosthetic aortic valve replacement who presented to the emergency department complaining of two days of diffuse abdominal pain started two days ago associated with nausea and vomiting. Vital signs were within normal limits except tachycardia with a heart rate of 117 and 101.5 F fever. He denied cough, chest pain, shortness of breath, lightheadedness, diarrhea, or constipation. No dysuria, urgency, or frequency was described. Regarding initial physical examination, the lungs were clear to auscultation. The heart rate was increased with a regular rhythm, no murmurs, rubs, or gallops heard. The abdomen was tender to palpation, bowel sounds were positive, and no significant distention, rebound, or guarding was found. He has a colostomy due to diverticulitis perforation and sigmoid colon resection one year ago. The colostomy was pink, soft, patent, and slightly edematous. There was also brown color stool in the bag. No signs of diarrhea or bleeding. The patient had poor dentition with several dental cavities. He has a history of congestive heart failure and valvular heart disease and underwent bioprosthetic aortic valve replacement with 27 mm pericardial tissue valve and mitral valve repair with 34 mm annuloplasty ring 16 months ago. He was found to have uncontrolled hyperglycemia with glucose level 536 mg/dL (normal: 70–115) with a bicarbonate level of 16 mEq/L (normal: 22–26) but no acidosis on arterial blood gas (ABG) with a pH of 7.4 (normal: 7.35–7.45). Urinalysis showed glucose >500 mg/dL (normal: negative) and ketones of 80 mg/dL (normal: negative). Hemoglobin A1c was 9.8 (normal: 4–6). Complete blood count showed leukocytosis with a white blood cell count of 11.000 cells/mcL (normal: 4.500–10.00), and basic blood workup was unremarkable. Electrolytes, lipase, liver, and kidney function tests were within normal limits. A chest X-ray showed an enlarged cardio-mediastinal silhouette without signs of infection. Electrocardiogram (ECG) revealed sinus tachycardia, occasional PVCs, and minimal ST depression noted. The troponin level was negative. IV fluids and insulin treatment initiated for the hyperglycemic hyperosmolar coma. Due to persistent abdominal pain and fever, a CT scan of the abdomen and pelvis with contrast was done, which showed herniation of the loop of the bowel at the colostomy site within the left upper pelvis and without evidence of bowel obstruction. Splenic infarct with lobulated large splenic abscess collection is seen within the spleen measuring 6.6 cm. The spleen is enlarged in size and measures 16.3 cm in length (Figure 1).

**Figure 2:**
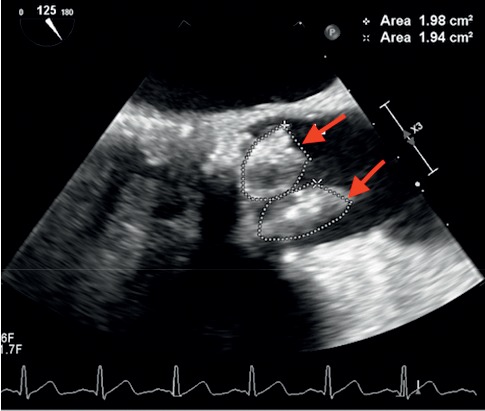
Transesophageal echocardiography showing two masses of approximately two cm^2^ each of mobile vegetations on the bioprosthetic aortic valve.

The following day, blood culture result was identified as *Lactobacillus acidophilus* in two sets of anaerobic bottles. Treatment with vancomycin 1750 mg IV twice a day, ceftriaxone 2 g IV daily, and metronidazole 500 mg IV 3 times a day was initiated. Considering the patient's previous history of IV drug use, bioprosthetic aortic valve replacement, and bacteremia, infective endocarditis was suspected. Transthoracic echocardiogram (TTE) was unremarkable, and a transesophageal echocardiogram (TEE) was performed. The report showed two masses of approximately two cm each of mobile vegetations on the bioprosthetic aortic valve (Figure 2). Normal overall left ventricular systolic function without regional wall motion abnormalities was observed, with an ejection fraction of 60%. Susceptibility testing showed that *Lactobacillus acidophilus* organism sensitive to clindamycin, erythromycin, and penicillin and resistant to vancomycin. The patient was transferred to a different facility to undergo replacement of the bioprosthetic aortic valve and drainage of the splenic abscess meanwhile based on in vitro susceptibility test antibiotheraphy replaced with clindamycin 2400 mg plus benzylpenicillin 24 million units. Upon transfer to another facility, the patient underwent a replacement of bioprosthetic aortic valve and abscess drainage procedures successfully. The patient will be on eight weeks of antibiotheraphy.

## 3. Discussion

*Lactobacillus* species rarely cause infection in humans, especially endocarditis, and splenic abscesses are exceptionally rarely encountered. *Lactobacillus acidophilus* is a commensal organism that has limited clinical relevance. Most of the time, identifying *Lactobacillus* species is challenging and can cause a delay in diagnosis and timely treatment. In general practice, *Lactobacillus* species isolated from blood cultures are not further characterized due to their perceived low virulence. *Lactobacillus* species have been reported as a cause of primary bacteremia, meningitis, liver, and splenic abscesses, and infective endocarditis. Endocarditis was diagnosed based on the modified Duke's criteria [11]. This present patient fulfilled two major criteria: new valvular regurgitation and persistently positive blood cultures in two different sets drawn >12 hours apart, and three minor criteria: fever, major arterial embolus which was splenic infarction, and injection drug use. Splenic abscess is also an uncommon but well-described complication of infective endocarditis. Abdominal CT scan and MRI are considered gold standard techniques for the diagnosis of the splenic abscess, with sensitivity and specificity varying between 90% and 95% [12]. In our patient, the diagnosis of the splenic abscess was based on the findings of the abdominal CT scan. In a series of 564 patients with documented endocarditis, the splenic abscess was reported in only 27 cases (4.8%) [13].

*Lactobacillus* species are identified in 0.05% to 0.4% of all endocarditis cases. [14] *Lactobacillus casei* is the most frequently identified species in the current literature, followed by *rhamnosus* and *plantrum* species [6]. *Lactobacillus acidophilus* is not commonly encountered compared with other species, and there are only a few previous cases of *Lactobacillus acidophilus* infective endocarditis reported in the literature [7–9]. Some specific conditions can be related to increased risk of *Lactobacillus bacteremia* and endocarditis, such as impaired immunity, structural heart disease, bioprosthetic valves, poor dentition, and severe comorbidities. Especially in patients who have significant underlying clinical conditions, physicians should consider *Lactobacillus* species as a causative microorganism. Our patient presented with uncontrolled diabetes mellitus and a history of bioprosthetic aortic valve replacement, IV drug abuse, and poor dentition. The most common source of Lactobacillus infections are oral mucosa, gastrointestinal, and genitourinary tracts. The source of the *Lactobacillus bacteremia* should be investigated because of the risk of mortality associated with *Lactobacillus endocarditis* was reported as high as 30% [6].

In a review of 129 cases of *Lactobacillus bacteremia*, Cannon et al. [6] noted malignancy as the most common underlying condition, followed by diabetes mellitus. Sussman et al. [15] reported that 83% of *Lactobacillus endocarditis* occurs in patients with preexisting structural heart disease, and 75% has recent dental infection or manipulation. In the present patient, poor dentition and bioprosthetic valve replacement were high likely the predisposing factors for *Lactobacillus endocarditis*. Cannon et al. [6] reported that dental procedures or poor dentition are the predisposing conditions in up to 50% of the *Lactobacillus*-induced endocarditis cases. Thus, the maintenance of oral hygiene is crucial in order to prevent systemic infections. Sources of Lactobacilli are likely from either gastrointestinal or genitourinary tracts. Husni et al. [16] reported that 38% of 45 cases with *Lactobacillus bacteremia* had underwent abdominal or endoscopic procedures preceding the bacteremia onset. Also, consuming probiotics was hypothesized to be a risk factor for Lactobacilli infections; however, no causative relationship was found [17].

A multispecialty heart valve team comprising infectious disease specialists, cardiothoracic surgeons, cardiologists, and general surgeons should manage patients with infective endocarditis and splenic abscess. Multidisciplinary treatment of infective endocarditis has been found to reduce mortality. *Lactobacillus bacteremia* and endocarditis are effectively treated via combination antibiotics, including beta-lactams and aminoglycosides for at least four weeks for native valve endocarditis and for up to eight weeks for prosthetic valve endocarditis. Salminen et al. [1] showed that in vitro susceptibility tests guided antibiotherapy choice significantly reduced mortality. In our case, susceptibility testing showed that the *Lactobacillus acidophilus* was sensitive to clindamycin, erythromycin, and penicillin, and resistant to vancomycin. Adequate antibiotic treatment should accompany surgery in the management of splenic abscess. The indications for surgery are the same as for other forms of endocarditis [18].

## 4. Conclusions

In conclusion, we report a rare condition by a rare organism. We believe that it will increase awareness regarding *Lactobacillus acidophilus* as possible sources of infection. The detection of *Lactobacillus bacteremia* should always prompt further investigation of its source and ensure clearance to prevent the high rate of mortality.
